# Comparative Study on the Surface Properties of Synthetic Carbonated Hydroxyapatite and Natural Hydroxyapatite Before and After Contact with Solutions with de- and Remineralization Activity

**DOI:** 10.3390/biomimetics11050338

**Published:** 2026-05-12

**Authors:** Radost Ilieva, Ivalina Avramova, Ognyan Petrov, Diana Rabadjieva

**Affiliations:** 1 Institute of General and Inorganic Chemistry, Bulgarian Academy of Sciences, Acad. G. Bonchev Str., bl.11, 1113 Sofia, Bulgaria; radipl@mail.bg (R.I.); iva@svr.igic.bas.bg (I.A.); 2 Institute of Mineralogy and Crystallography, Bulgarian Academy of Sciences, Acad. G. Bonchev Str., bl.107, 1113 Sofia, Bulgaria; opetrov52@gmail.com

**Keywords:** hydroxyapatite, demineralization, remineralization, XPS study, SEM study

## Abstract

Understanding the differences between synthetic and natural hydroxyapatite under conditions that mimic the oral environment, particularly the demineralization and remineralization processes of dental enamel, is essential for assessing their suitability as enamel models in biomineralization studies. The present study aims to systematically compare the structural, chemical, and morphological properties of well-crystallized synthetic carbonated hydroxyapatite (CHA) and natural non-biogenic hydroxyapatite (HA) before and after exposure to solutions with demineralizing and remineralizing activity. Two highly informative surface characterization techniques—X-ray photoelectron spectroscopy (XPS) and scanning electron microscopy (SEM)—were employed to examine the resulting surface changes. In addition, powder X-ray diffraction and infrared analyses were used to characterize the initial samples. Demineralization was induced using a lactic acid-based solution, while remineralization was performed through a two-step treatment involving polycarboxybetaine followed by artificial saliva. The results show that natural HA contains an additional fluorapatite phase and a wider range of trace elements (Na, F, Si), leading to a more complex structure. During demineralization, synthetic CHA exhibits more pronounced surface changes and faster dissolution, whereas natural HA demonstrates greater chemical stability. The remineralization process leads to the formation of new surface layers on both materials. Synthetic CHA develops a fine-grained, homogeneous layer enriched in carbonate and hydrated species, while natural HA shows localized crystal growth within structural defects. The results demonstrate that natural HA exhibits greater chemical stability during demineralization and a more enamel-like response during remineralization, whereas synthetic CHA undergoes more pronounced surface restructuring and forms a highly hydrated, carbonate-rich surface layer.

## 1. Introduction

Hydroxyapatite (HA, Ca_10_(PO_4_)_6_(OH)_2_) is a calcium phosphate compound with chemical and structural similarity to the inorganic constituent of human and animal bones and teeth, making it a suitable biomaterial in orthopedics, dentistry, and tissue engineering. In addition, HA exhibits excellent biocompatibility, osteoconductivity, and chemical stability, which determine its wide applications [1,2]. To be used in medicine, HA must have not only chemical but also structural properties similar to those of the hard human tissues with which it would be in contact. In dentistry, enamel consists of over 95% crystalline HA, with crystals highly oriented along the long axis of the tooth, forming organized structures known as enamel prisms or rods [3]. This oriented crystalline arrangement contributes significantly to enamel’s hardness and its wear resistance [4]. Therefore, HA, resulting from enamel remineralization, must reproduce not only the composition but also, as far as possible, the structural organization of natural enamel in order to achieve comparable properties.

Remineralization is the natural and therapeutic process by which damaged enamel is restored, strengthening teeth and preventing progression to deep caries. Enamel demineralization occurs when acids produced by bacterial metabolism lower the oral pH below 5.5, dissolving the enamel’s mineral structure [5,6]. If the acidity is neutralized and mineral ions are available, the enamel can be restored and early enamel lesions can be partially reversed [7,8,9]. Natural saliva contains calcium and phosphate ions [10]; however, external sources of mineral ions are applied to accelerate the process [11,12,13]. Typically, solutions containing lactic or citric acid are used to create artificial lesions in enamel and simulate demineralization processes [14], while different remineralizing agents, including fluoride, bioactive glass, and nano-hydroxyapatite, have been widely investigated in combination with artificial saliva to evaluate enamel remineralization [15,16].

Synthetic HA dominates clinical use because it combines reproducibility, high purity, and tunable physicochemical properties [17,18,19]. This strategy for enamel remineralization still faces the challenge of achieving oriented, well-arranged calcium phosphate apatite crystals on the enamel surface. Even the biomimetic approach involving directed precipitation by organic macromolecules still yields only moderate results [20].

HA derived from natural sources—such as bovine bone, fish bone, eggshells, or corals—has also attracted interest for dental remineralization. After high-temperature purification, natural biogenic-source HA retains carbonate, magnesium, and strontium substituents that enhance bioactivity and promote crystal growth on demineralized enamel [21,22].

Natural non-biogenic (geological) HA, found in phosphate rocks and mineral veins, exhibits higher crystallinity, larger crystallite size, and lower solubility than synthetic and biogenic HA, often with carbonate or fluoride substituents [23]. Apatite, Ca_5_(PO_4_)_3_(Cl, F, OH), is the most common rock-forming phosphate mineral, often occurring as well-formed hexagonal crystals, as in Sakar Mountain, Bulgaria [24].

In this work, we hypothesize that well-formed crystals of natural HA can serve as a model for the ordered structure of dental enamel when studying biomineralization processes or when evaluating novel remineralizing systems. As a continuation of our previous study [25], the present work aims to compare the surface properties of synthetic well-crystallized carbonated and natural non-biogenic HA before and after exposure to solutions with demineralizing and remineralizing activity under conditions that approximate those in the oral cavity. A lactic acid solution was selected as the demineralizing agent because it mimics the acidic conditions that arise in the oral environment during the bacterial metabolism of dietary sugars. The remineralization process was carried out in two steps. Initially, a polycarboxybetaine solution was applied to stimulate the materials for the subsequent remineralization step when treated with artificial saliva. To examine the resulting surface changes, two highly informative surface characterisation techniques—X-ray photoelectron spectroscopy (XPS) and scanning electron microscopy (SEM)—were employed.

There are comparisons in the literature of the properties of synthetic and natural HA, but mainly of biological origin. In recent years, Montesissa et al. [26] compared nanostructured coatings created using ionized jet deposition from synthetic HA and natural sources, including bovine, equine, and porcine bone. They found that coatings from natural sources are more biomimetic, containing trace ions (Mg, Na), and showed superior stability in liquid medium, while synthetic coatings exhibited cracking and detachment after 7 days of exposure. Amaechi et al. [27] conducted an in vitro study to compare the remineralization effectiveness of dentifrices containing natural (obtained from biowaste sources such as bones, fish scales, and eggshells) and synthetic HA, and standard fluoride toothpaste on initial enamel caries lesions. Synthetic HA has an ideal calcium-to-phosphate ratio (1.67), but it lacks beneficial trace elements. In contrast, natural HA contains trace ions such as magnesium, sodium, and strontium, making it more similar to human enamel and dentin. Despite these differences, both types demonstrate comparable ability to remineralize early caries lesions by supplying calcium and phosphate ions and filling demineralized areas of enamel.

To our knowledge, a systematic comparison of the surface behavior (using XPS) of synthetic CHA and natural non-biogenic (geological) HA before and after exposure to solutions with demineralizing and remineralizing activity has rarely been considered in the literature. To fill this gap, the present study aims to use a combination of XPS and SEM techniques to provide new insights into the surface transformations of the materials under study and to evaluate them as model systems for enamel de- and remineralization studies.

## 2. Materials and Methods

### 2.1. Chemicals Used

Natural HA was provided by the Earth and Man National Museum, Bulgaria. Its characterization is presented in Section 3.1.

The following substances were used to prepare the stock solutions for synthesizing synthetic CHA and to prepare the solutions that possess de- and remineralization activity: Ca(NO_3_)_2_ (Sigma-Aldrich, St. Louis, MO, USA, 99%), (NH_4_)_2_HPO_4_ (Sigma-Aldrich, St. Louis, MO, USA, 98%), NH_4_OH (Charlotte, North Carolina, USA, 25% NH_3_ in H_2_O), lactic acid (Sigma-Aldrich, St. Louis, MO, USA, 85%), NaH_2_PO_4_ (Sigma-Aldrich, St. Louis, MO, USA, 98%), CaCl_2_·2H_2_O (Sigma-Aldrich, St. Louis, MO, USA, 99%), NaF (Sigma-Aldrich, St. Louis, MO, USA, 99%), NaCl (INEOS, A.R., London, UK, 99%), KCl, (INEOS, A.R., London, UK, 99%), K_2_HPO_4_·3H_2_O (Sigma-Aldrich, St. Louis, MO, USA, 98%), Na_2_HPO_4_ (Sigma-Aldrich, St. Louis, MO, USA, 98%), NaSCN (Sigma-Aldrich, St. Louis, MO, USA, 98%), NH_4_Cl (Reanal, Budapest-Hungary, 99%), urea (Sigma-Aldrich, St. Louis, MO, USA, 99%), glucose (Sigma-Aldrich, St. Louis, MO, USA, 99.5%), ascorbic acid (Sigma-Aldrich, St. Louis, MO, USA, tested according to Ph. Eur.) and mucin from porcine stomach Type II (Sigma-Aldrich, St. Louis, MO, USA). The preparation of polycarboxybetaine (PCB) is by a method described in the literature [28].

### 2.2. Preparation of Synthetic Well-Crystallized CHA

A two-step procedure was applied: (i) precursor precipitation and (ii) high temperature calcination. The wet method of continuous precipitation was applied for precursor synthesis. Solution of Ca(NO_3_)_2_ (1 mol·L^−1^) was added to a solution of (NH_4_)_2_HPO_4_ (0.6 mol·L^−1^) at a Ca/P ratio of 1.67, pH 2 (maintained with NH_4_OH) and stirring on an overhead stirrer (IKA^®^ RW 16 basic, IKA^®^-Werke GmbH & CO. KG, Staufen im Breisgau, Germany) at a speed of 350 rpm for 2 h. The resulting precipitate was matured for 24 h without stirring, centrifuged (2000 rpm for 10 min for each portion) using Sigma 2–7 centrifuge (Osterode am Harz, Germany), washed three times with water by centrifugation (2000 rpm for 10 min), and dried at 90 °C for 15 h. The obtained powder was poorly crystalline HA (See Supplementary Material, Figure S1).

To prepare well-crystallized HA, the dried powder from poorly crystalline HA was milled for 3 h at 500 rpm in a ball mill (Fritsch 6, Fritsch GmbH, Idar-Oberstein, Germany) and sifted through a 0.07 mm sieve. A 0.65 g sample of sieved powder was pressed into tablets at 7 tons for 2 min using a SPECAC GS15011 press (Specac Ltd., Orpington, UK). The tablets were subsequently calcined at 1000 °C in air in a high-temperature furnace (VP 04/17, LAC Ltd., Rajhrad, Czech Republic) with a heating rate of 10 °C·min^−1^ and a 1 h hold. The tablet dimensions were 13 mm in diameter and 2.29 mm in height. Characterization of the calcined material is presented in Section 3.1.

### 2.3. Preparation of Tablets from Natural HA

The tablets of natural, non-biogenic HA were prepared using an ATM-Brillant 220 (ATM Qness GmbH, Mammelzen, Germany) precision cutting device.

The tablets were 13 mm in diameter and 2.29 mm high. Characterization of the HA is presented in Section 3.1.

### 2.4. Demineralization Process

The demineralization process was carried out using a solution composed of 0.1 mol·L^−1^ lactic acid, 2.2 mmol·L^−1^ sodium dihydrogen phosphate, 2.2 mmol·L^−1^ calcium dichloride, and 0.005 mmol·L^−1^ sodium fluoride, with the pH set to 4.5. This solution has been used in previous studies to simulate artificial lesions on extracted teeth [28,29]. Each tablet was immersed in 70 mL of the demineralizing solution in a sealed plastic container positioned vertically to ensure uniform exposure to the solution volume. Only the bottom side of the tablets was evaluated to prevent the influence of incidental factors, such as mineral deposition from sedimentation, on the top surface. The experiments were conducted under static conditions (without stirring) at room temperature for durations of 6 and 12 h for both sample sets. Given the established stability of natural HA after a 12 h exposure to a demineralization solution, experiments with it were conducted for 6 days, which is the exposure duration of dental specimens in the methodology for obtaining artificial lesions in tooth enamel [28,29].

### 2.5. Remineralization Process

The remineralization process was performed using the same experimental setup (one tablet with dimensions of 13 mm in diameter and 2.29 mm in height, and 70 mL of solution) and conditions as applied in the demineralization stage. The protocol followed a two-step approach previously developed in our study on the remineralization activity of hybrid materials on artificially induced enamel lesions in extracted tooth samples [28,29].

In the first step, the tablet surfaces were biostimulated for 6 h in a solution containing 0.05% PCB, 0.05% calcium dichloride, and 0.023% dipotassium hydrogen phosphate. Polycarboxybetaine is a zwitterionic polymer known for promoting conditions favorable for mineral nucleation [28,29].

Subsequently, the biostimulated tablets were immersed for 18 h in artificial saliva prepared according to Klimek [30], with the following composition: 9.92 mmol·L^−1^ sodium chloride, 17 mmol·L^−1^ potassium chloride, 1.5 mmol·L^−1^ calcium dichloride, 2.42 mmol·L^−1^ dipotassium hydrogen phosphate, 3.03 mmol·L^−1^ disodium hydrogen phosphate, 1.98 mmol·L^−1^ sodium tiocianate, 2.99 mmol·L^−1^ ammonium chloride, 3.33 mmol·L^−1^ urea, 0.17 mmol·L^−1^ glucose, 0.01 mmol·L^−1^ ascorbic acid, and 2700 mg·L^−1^ mucin.

This 24 h cycle was repeated for 6 days.

### 2.6. Characterizations

Powder X-ray diffraction (PXRD) analysis was conducted using a Bruker D8 Advance diffractometer (Bruker AXS Advanced X-ray Solutions GmbH, Karlsruhe, Germany). CuKα radiation (λ = 0.15418 nm) was employed as the X-ray source, and diffraction patterns were recorded using a LynxEye detector (Bruker AXS Advanced X-ray Solutions GmbH, Karlsruhe, Germany). Data were collected over a 2θ range of 10–90°, with a step size of 0.03° and a counting time of 57 s per step for phase identification.

X-ray photoelectron spectroscopy (XPS) measurements were carried out using an Axis Supra electron spectrometer (Kratos Analytical Ltd., Manchester, UK) equipped with a monochromatic AlKα source (1486.6 eV). Binding energies (BE) were calibrated against the C1s peak at 285.0 eV, with an accuracy of ±0.1 eV. Photoelectron spectra of the surface elements were acquired, background-corrected using a Shirley-type function, and quantified based on peak areas and Scofield photoionization cross-sections. Spectral deconvolution, when required, was performed using XPSPEAK41 software, version 4.1. To evaluate potential surface heterogeneity, spectra were collected from two distinct regions of each sample. As no notable differences were detected, only the spectra and corresponding quantitative results from a representative area are reported.

Scanning electron microscopy (SEM) analysis was performed using a JEOL JSM 6390 (JEOL Ltd., Akishima (Tokyo), Japan) instrument to examine the morphology of the initial, demineralized, and remineralized tablet surfaces. Before imaging, the samples were sputter-coated with gold for 40 s under vacuum, and micrographs were acquired at various magnifications.

Infrared (IR) spectroscopy was performed using an IRAffinity-1 Fourier Transform Infrared (FTIR) spectrophotometer (Shimadzu Co., Kyoto, Japan) equipped with a MIRacle™ reflection accessory ( PIKE Technologies, Inc., Madison, WI, USA). The vibrational spectra were collected in the 400–4500 cm^−1^ range directly on the material, without any prior sample preparation.

## 3. Results

### 3.1. Characterization of the Initial Synthetic CHA and Natural HA

**Figure 1:**
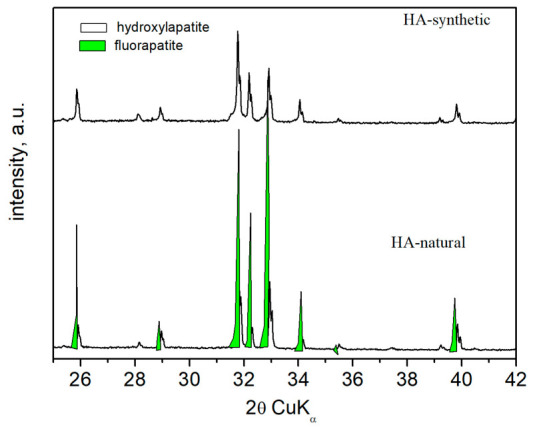
PXRD powder pattern of the natural apatite sample and that of the synthesized material.

The PXRD pattern (Figure 1) shows crystallized HA in both samples.

In the PXRD pattern of natural HA (HA-natural), the strongest diffraction lines are split. Inspecting the additional diffraction lines with a search in the database PDF confirmed a second apatite phase—fluorapatite (Ca_5_(PO_4_)F). However, the lines of fluorapatite are narrower, suggesting larger microcrystals than those of HA, which are broader, indicating smaller crystallites. The synthesized material (sample HA-synthetic) is characterized by the presence of only the HA phase. These XRD peaks are split (α_1_**/**α_2_ doublet), indicating well-crystallized hydroxyapatite micro-crystals.

**Figure 2:**
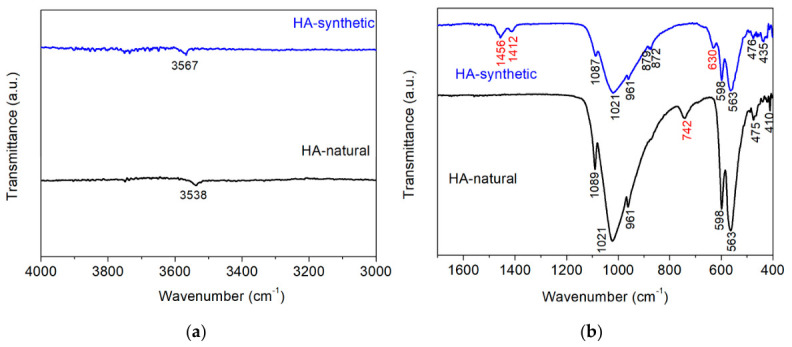
IR spectra of (**a**)—natural and synthetic CHA in the region of 4000–3000 cm^−1^ and (**b**)—1700–400 cm^−1^.

The FTIR spectra of both samples (Figure 2) confirm the formation of an apatite calcium phosphate structure, as evidenced by the characteristic phosphate (PO_4_^3−^) vibrational modes reported for crystalline apatite [31,32]. In both natural and synthetic apatite, the bands at ~1086–1089 and 1020–1021 cm^−1^ correspond to the ν_3_ asymmetric stretching of PO_4_^3−^, while the band at ~961 cm^−1^ is assigned to the ν_1_ symmetric stretching mode. The ν_4_ bending modes appear at ~598 and 563–565 cm^−1^, and the ν_2_ bending modes are observed near 475–464 cm^−1^, confirming a crystalline apatite framework in both materials.

Clear differences arise from hydroxyl and carbonate-related vibrations. The synthetic material exhibits a sharp OH stretching band at 3567 cm^−1^ (Figure 2a), together with a distinct OH libration band at approximately 630 cm^−1^ (Figure 2b), characteristic of hydroxyl-rich hydroxyapatite [32]. In contrast, the natural apatite shows a weaker and slightly shifted OH stretching band at ~3538 cm^−1^ (Figure 2a) and lacks a clear librational band, indicating partial substitution of the OH^−^ group.

The synthetic sample also displays pronounced carbonate bands at 1456 and 1412 cm^−1^ (ν_3_ CO_3_^2−^) and at 879–872 cm^−1^ (ν_2_ CO_3_^2−^), which are typical for B-type carbonate substitution, where CO_3_^2−^ replaces PO_4_^3−^ in the apatite lattice [33,34] (Figure 2b). Therefore, the synthetic material will be hereinafter referred to as synthetic carbonate hydroxyapatite (CHA). These carbonate bands are absent in the natural apatite, suggesting a carbonate-poor structure. The natural apatite additionally exhibits a band at approximately 742 cm^−1^, attributed to external phosphate lattice vibrations, which is absent in the synthetic sample and reflects enhanced lattice ordering and probably fluoride stabilization associated with the fluorapatite component [32].

**Figure 3:**
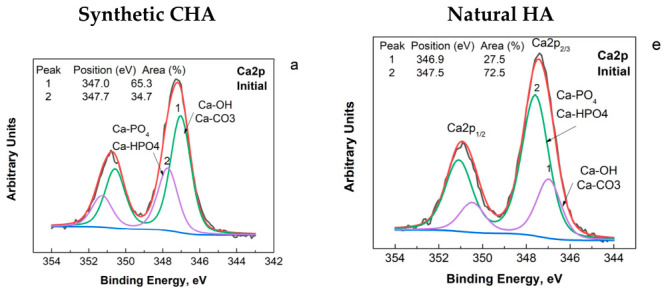

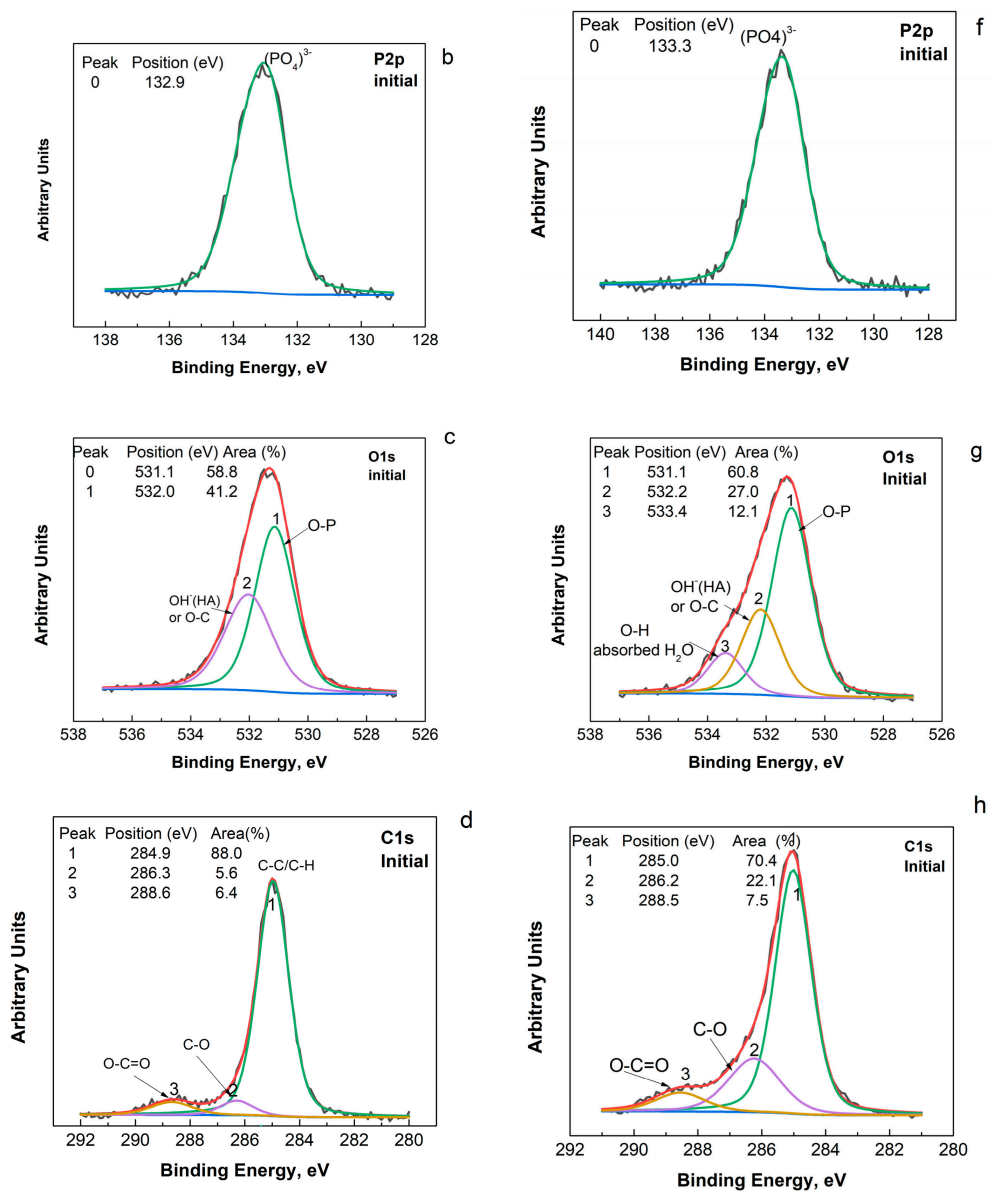
XPS high-resolution spectra for Ca2p (**a**,**e**); P2p (**b**,**f**), O1s (**c**,**g**) and C1s (**d**,**h**) of the synthetic CHA (**a**–**d**) and HA natural (**e**–**h**).

XPS analysis of the elementary composition (Table 1) and XPS spectra (Figure 3) of the samples under study shows that the surface of both samples is composed of Ca, P, O, and C, confirming that they share a common structure. Oxygen and carbon are the dominant elements in all materials, indicating the presence of phosphate and carbonate groups. In addition, the Ca/P ratio (Table 1) is close to the expected range for apatite-type materials in all cases, although it varies significantly among the samples. However, their chemical compositions differ markedly. Synthetic CHA shows a Ca/P ratio of 2.36, which is higher than that of stoichiometric HA (1.67), natural HA (1.51), and enamel (1.31), suggesting a more calcium-rich and less stoichiometric structure on the surface. The higher Ca/P ratio is associated with the presence of CO_3_ groups replacing PO_4_^3−^ groups in the structure or impurities of CaCO_3_. All of this corresponds to the high surface C content. This ratio suggests a higher solubility of the material in biological media [35]. Natural HA contains a wider variety of trace elements, including Na, F, N, and Si, which are absent in synthetic CHA. This reflects the more complex formation environment of natural HA. Enamel contains a relatively high amount of nitrogen and shows the highest O/P ratio (10.4), indicating a different balance between components.

The binding energies (Figure 3) of Ca2p, P2p and O1s are in agreement with those of HA or other phosphates [37,38,39]. In the Ca2p region, both materials (Figure 3a,e) exhibit the characteristic doublet corresponding to Ca2p_3_/_2_ and Ca2p_1_/_2_, confirming the presence of calcium in a comparable chemical state in both HA samples. The Ca2p binding energy positions are very close, with only minor shifts (Figure 3a,b), which can be attributed to slight variations in the local electronic environment of Ca, arising from differences in crystallinity, lattice disorder, or surface chemistry rather than from fundamentally different Ca coordination.

Differences in the relative intensities and areas of the Ca2p doublet components are observed in Figure 3a and Figure 3b, respectively. The higher contribution (65.3% area, Figure 3a, peak 1) of the Ca2p component associated with carbonate-bound environments in synthetic CHA suggests a greater presence of surface calcium carbonate or carbonate-substituted species. In contrast, calcium predominantly bound in phosphate environments is more prevalent on the surface of natural HA (72.5% area, Figure 3b, peak 2). The P2p region (Figure 3b,f) further emphasizes the chemical similarity of the two materials.

More pronounced differences are observed in the O1s (Figure 3c,g) spectra. Synthetic CHA shows two components, usually associated with lattice oxygen (peak 1) and hydroxyl or adsorbed oxygen species (peak 2). Natural HA displays three components. The broader and asymmetric O1s envelope in natural HA indicates a more complex oxygen chemistry, likely arising from structural water, carbonate substitution, or surface hydroxylation.

Carbon is also a major element detected on the surface, which can result from surface adsorption processes as well as from the HA origin. In the case of the synthetic CHA, the presence of surface carbon can be attributed to synthesis and processing in open air, which promotes adsorption of atmospheric carbon species. For natural HA, the carbon signal may also reflect carbonate substitution in the apatite lattice and residual organic matter associated with the material’s origin. The C1s spectra (Figure 3d,h) of both materials exhibit three deconvoluted components attributed to C–C/C–H bonds, C–O groups, and O–C=O or carbonate species. Although the binding energies are very similar for both samples, differences in the relative intensities of these components suggest variations in surface carbon chemistry.

**Figure 4:**
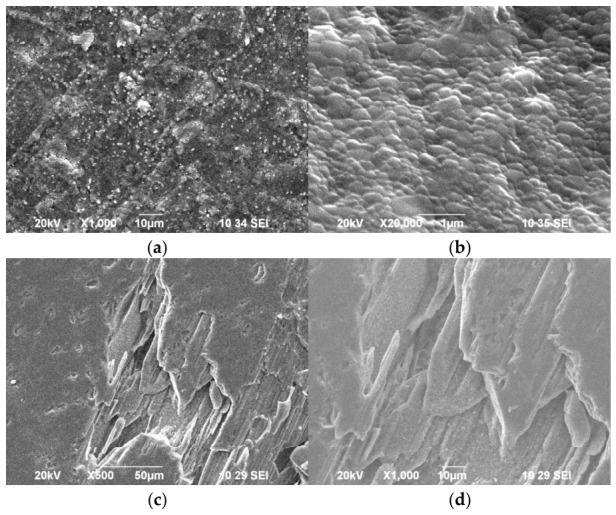
SEM images of synthetic CHA (**a**,**b**) and natural HA (**c**,**d**). The selected magnifications are tailored to the size of the crystals in the samples.

SEM images (Figure 4) also highlight the morphological differences between synthetic CHA and natural HA.

The surface of synthetic CHA (Figure 4a) is characterized by a dense, granular texture with small, relatively uniform grains tightly packed next to each other, which is typical of synthetic CHA. On the surface of the natural HA, two types of areas are identified (Figure 4b,c). Figure 4b shows an uneven and porous surface with fine particles, highlighting the complex topography often found in biological minerals. Figure 4c reveals a distinct layered or plate-like morphology, suggesting a more hierarchical structural organization.

### 3.2. Effect of Exposure to a Solution with Demineralization Activity

Tablets of both materials were exposed to a lactic acid-based solution, known for its demineralizing activity.

**Figure 5:**
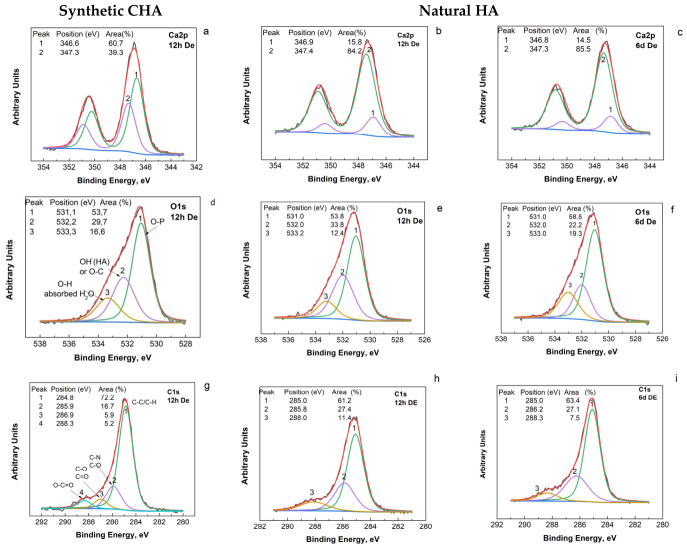
XPS high-resolution spectra for Ca2p (**a**–**c**); O1s (**d**–**f**) and C1s (**g**–**i**) of the synthetic CHA after 12 h contact with solution with demineralization activity (**a**,**d**,**g**) and HA natural HA after 12 h contact with solution with demineralization activity (**b**,**e**,**h**) and after 6 days contact with solution with demineralization activity (**c**,**f**,**i**). P2p spectra are not included, as no differences relative to the initial spectra were observed.

A comparison of the XPS high-resolution spectra of synthetic CHA before and after 12 h of exposure to the lactic acid–based demineralizing solution showed no differences in the Ca2p region, either in the positions of the doublets or in their relative intensities (Figure 3a and Figure 5a).

In contrast, noticeable alterations were observed in the O1s and C1s regions. The deconvoluted O1s spectra show the appearance of a component attributed to adsorbed water (Figure 3c and Figure 5d), while the C1s spectra show a component that relates to the chemical bond between carbon and nitrogen or oxygen (C-O, C-N) and can be attributed to the effect of the environment (Figure 3d and Figure 5g). In comparison, natural HA exhibited no detectable changes in the spectra of its main constituent elements (Ca, P, O, and C), neither after 12 h nor after 6 days of contact with the solution. As mentioned in Section 2.4, 6 days corresponds to the exposure time of dental specimens in the methodology for obtaining artificial lesions in tooth enamel [28,29].

Quantitative analysis also shows greater changes for synthetic CHA (Table 1). The Ca, P, and O contents increased to 8.6, 5.2, and 33.3 at.%, respectively, while the carbon content decreased to 52.3 at.%. The Ca/P ratio decreased to 1.65, approaching the stoichiometric value. In contrast, natural HA exhibited smaller compositional variations, with Ca/P ratios of 1.36 after 12 h and 1.38 after 6 days. The relative concentrations of C and O fluctuated slightly, but the overall composition remained close to the initial sample. Minor elements such as Na, F, N, and Si were preserved.

**Figure 6:**
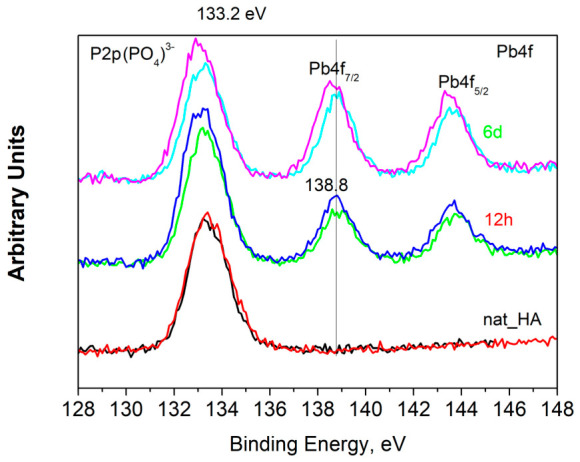
XPS high-resolution spectra for Pb4f of initial natural HA (nat_HA) and after 12 h and 6 days contact with solution with demineralization activity. Identification according to Lopez et al. [39]. The two colors for each sample indicate measurement at two different locations.

Pb appeared in the chemical composition (Table 1, Figure 6). The Pb 4f_7/2_ peak (Figure 6), centered at 138.6 eV, is consistent with the Pb 4f_7/2_ binding energies of highly ionic compounds such as Pb(OH)_2_ (138.2 eV), PbCO_3_ (138.3 eV) and the HA isostructural chloropyromorphite Pb_5_(PO_4_)_3_Cl (138.9 eV) [39]. The presence of Pb may be due to incorporation into the mineral structure or to contamination by the reagents used to prepare the demineralization solution. Since the concentrations of the solution components are very low (see Section 2.4), the latter possibility is less likely. On the other hand, HA is known to be a very good sorbent of Pb from Pb-containing waters, whereby Pb^2+^ ions can replace Ca^2+^ ions [40].

**Figure 7:**
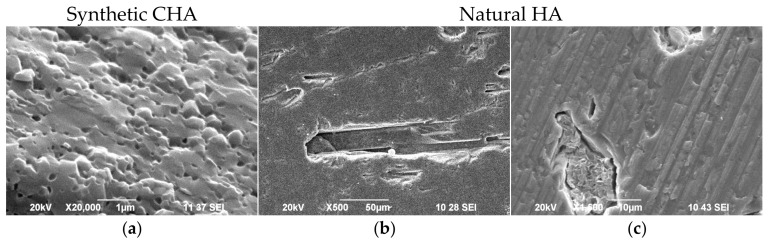

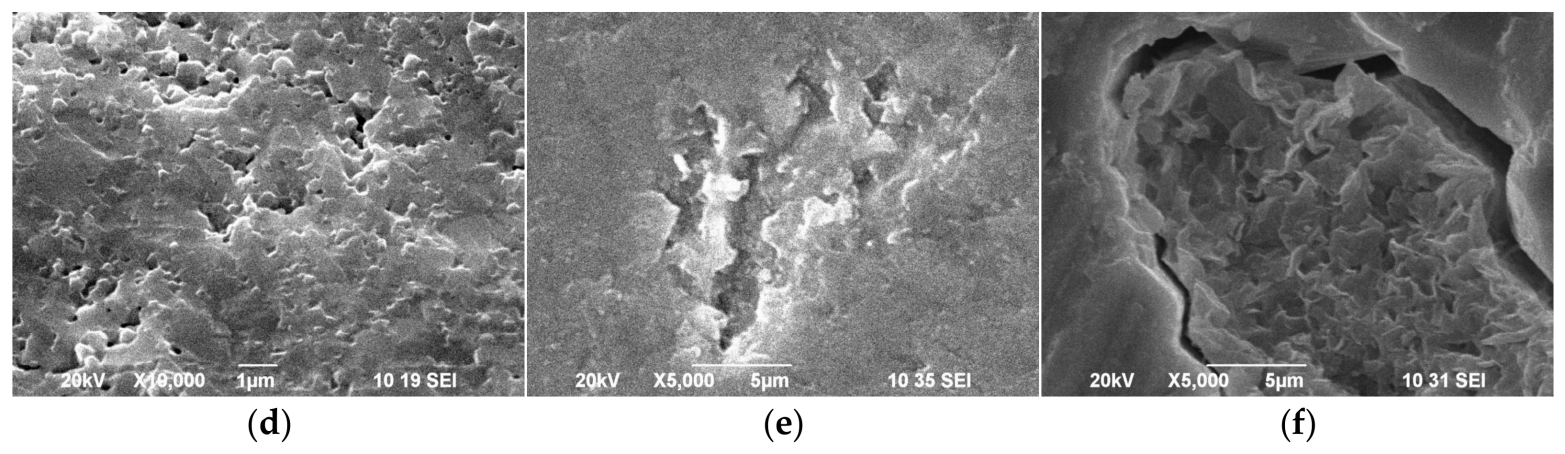
SEM images of the surface of synthetic CHA ((**a**)—6 h, (**d**)—12 h) and natural HA ((**b**)—12 h, (**c**)—6 d, (**e**)—12 h, (**f**)—6 d) after staying in solution with demineralization activity for different periods. h denotes hours, d denotes days. Images at different magnifications are presented for natural HA due to surface heterogeneity.

SEM images (Figure 7) also reveal differences between synthetic CHA and natural HA after exposure to a demineralization solution.

The surface of synthetic CHA undergoes significant changes, specifically the formation of pits on the grain surface during the initial 6 h of demineralization. By the 12 h mark, the surface appears smoother, with the sharp boundaries previously visible between individual crystals becoming less distinct.

Dissolution of the natural HA is scarcely detectable up to 12 h, when the first signs of surface degradation, including narrow cracks and slight pitting, begin to appear. After 6 days, this develops into pronounced destruction of the surface layer, with the attack primarily localized along inter-crystallite regions, thereby exposing the internal architecture as the solution penetrates deeper into the structure.

### 3.3. Effect of Exposure to a Solution with Remineralization Activity

A two-step remineralization procedure, as described in our previous studies [25,28], was applied to the surfaces of synthetic CHA after 12 h of exposure to a demineralizing solution and to natural HA after 6 days of exposure. The first step is to treat the demineralized surface with a solution of polycarboxybetaine (PCB), CaCl_2_ and K_2_HPO_4_. The results of the XPS analysis (Figure S2, Supplementary Materials) show no changes in the qualitative composition, but rather only small changes in the quantitative characteristics (area in %) of the deconvoluted peaks compared to those of the demineralized surfaces (Figure 5). This facilitates the formation of nucleation sites that control the growth of a new calcium phosphate phase. Afterward, the biostimulated tablets were incubated for 18 h in artificial saliva with a composition according to Klimek [30].

**Figure 8:**
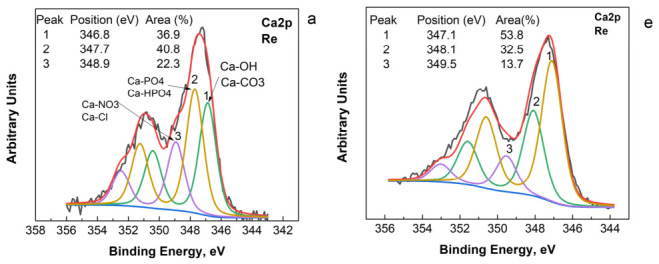

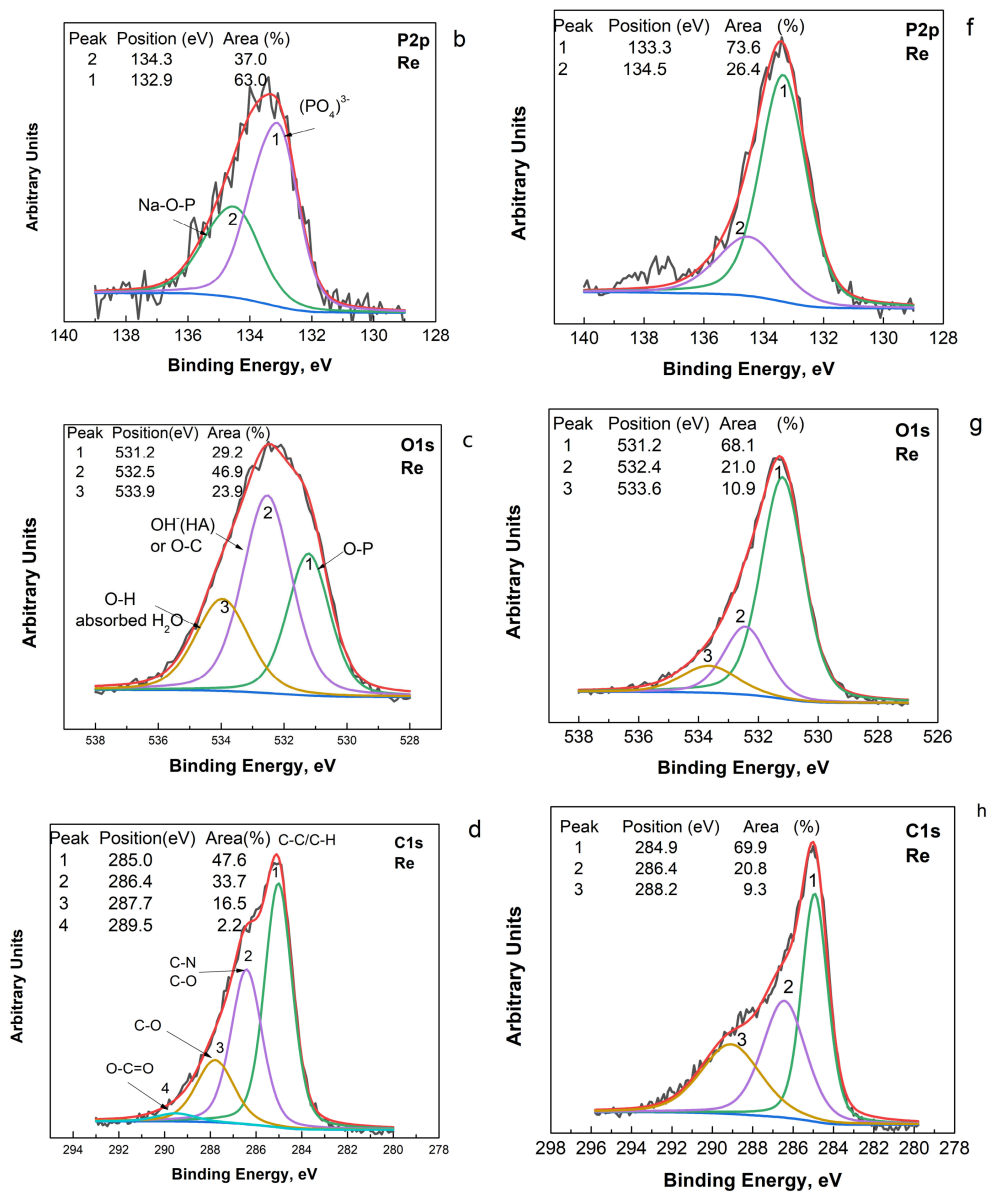
XPS high-resolution spectra for Ca2p (**a**,**e**), P2p (**b**,**f**), O1s (**c**,**g**) C1s (**d**,**h**) of the synthetic CHA and natural HA.

The remineralization treatment results in noticeable changes in the XPS spectra of Ca, P, O, and C of both materials (Figure 8).

The peaks become broader and more asymmetric, particularly for synthetic CHA, indicating modifications to the surface microenvironment due to interactions with the remineralizing solutions. Additional components appear in the deconvoluted Ca2p and P2p spectra, which can be attributed to newly formed chemical environments such as Ca–NO_3_/Ca-Cl (Figure 8a, peak 3) and Na–O–P (Figure 8b, peak 2) bonds. In addition, the relative contributions of phosphate- and carbonate-related calcium environments change in both samples: the carbonate-related component increases in natural HA (Figure 8e, peak 1), whereas the phosphate-related component becomes more pronounced in synthetic CHA (Figure 8a, peak 2). Furthermore, the relative intensities and area, respectively of the O–H(C–O) and C–O(C–N) bonding states increase in the deconvoluted O1s and C1s spectra, indicating a greater contribution of hydroxyl and organic-related species on the surface after remineralization.

Changes in the quantitative elemental composition are observed for both synthetic and natural HA (Table 1). However, the extent and nature of these changes differ significantly between the two materials. For synthetic CHA, remineralization leads to a pronounced decrease in the surface concentrations of calcium and phosphorus (Ca = 3.5 at.%, P = 2.1 at.%) compared with both the initial and demineralized states. At the same time, the carbon content increases substantially (70.0 at.%), while sodium also becomes more pronounced (0.9 at.%). These changes suggest the formation of a surface layer enriched in carbonate and organic species originating from the remineralizing medium. The Ca/P ratio after remineralization (1.72) increases relative to the demineralized surface (1.65), indicating partial reorganization of the calcium phosphate environment. In addition, the O/Ca and O/P ratios increase markedly (6.7 and 11.4, respectively), which may reflect the incorporation of hydroxyl- and carbonate-containing surface species and the development of a more hydrated surface layer. In contrast, natural HA demonstrates a more stable elemental composition after remineralization. The calcium and phosphorus concentrations remain relatively high (Ca = 7.3 at.%, P = 5.8 at.%), and the carbon content (49.9 at.%) is considerably lower than that observed for synthetic CHA. The Ca/P ratio decreases to 1.25, approaching the values reported for natural enamel. The O/Ca and O/P ratios (5.55 and 4.46, respectively) indicate a surface composition that remains closer to the natural mineral phase. Pb was no longer detected on the surface. This may be due to the formation of a new surface layer that effectively covered the surface sites where Pb was previously present, or to the removal of Pb by the remineralization solution. Our attempts to detect Pb^2+^ ions in the solution were unsuccessful.

**Figure 9:**
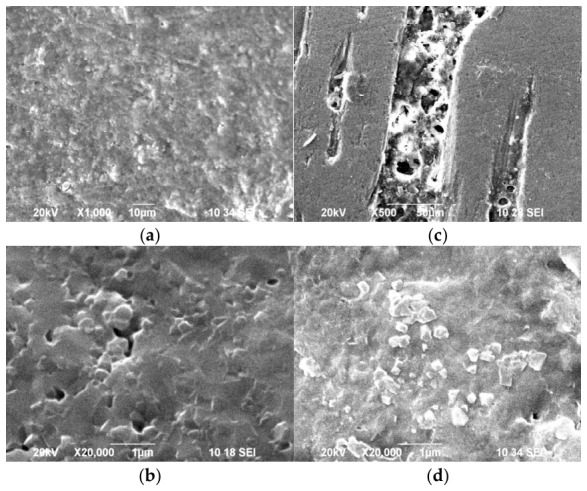
SEM images with different magnifications of the surface of synthetic CHA (**a**,**b**) and natural HA (**c**,**d**) after staying in solution with remineralization activity. The selected magnifications are tailored to the size of the crystals in the samples.

The formation of a new mineral phase on the surface of demineralized HA is also confirmed by SEM images (Figure 9). The remineralization of the synthetic material results in a dense and uniform coverage of the entire surface. The crystallites formed are significantly smaller than those observed in natural HA, resulting in a fine-grained, homogeneous texture. Remineralization of natural HA is characterized by the deposition of larger crystals with distinct geometric shapes. At high magnification (X20,000), these crystals appear as isolated, prominent structures scattered across the surface. At lower magnification (X500), it is evident that these deposits preferentially form within structural defects that were previously exposed during the dissolution phase.

## 4. Discussion

The combined structural, spectroscopic, and microscopic analyses performed in this study reveal significant differences in the surface properties and reactivity of synthetic CHA and natural non-biogenic HA. These differences arise primarily from their origin and structural complexity.

The PXRD, FTIR and XPS analyses (Figure 1, Figure 2 and Figure 3, Table 1) show that both materials possess an apatite calcium phosphate structure. However, while the synthetic sample consists of pure HA, a fluorapatite phase is identified in the natural material (Figure 1 and Figure 2). The FTIR spectra further highlight differences in lattice substitutions (Figure 2). The strong OH bands in synthetic CHA indicate a hydroxyl-rich structure, whereas the weaker and shifted OH band in natural HA suggests partial substitution of OH^−^ groups, likely by fluoride ions [41,42]. Moreover, the synthetic CHA exhibits stronger carbonate bands corresponding to B-type carbonate substitution, whereas carbonate vibrations are less evident in the natural sample, indicating different substitution patterns and lattice chemistry. Additionally, XPS analyses (Figure 3) show that natural HA exhibits broader, more asymmetric peaks, with differences in the intensities of their deconvoluted constituents, reflecting greater structural complexity, compositional heterogeneity, and the dominance of phosphate over carbonate groups in the CA environment. All of this is responsible for the higher chemical stability of natural HA observed during its stay in solution with demineralization activity. It is known that fluoride substitution stabilizes the apatite lattice and reduces its solubility, while carbonate ions increase it [43].

The experiments with the demineralization-active solution clearly demonstrate the different chemical stabilities of the two materials. Synthetic CHA undergoes noticeable surface modification after relatively short exposure to the lactic acid solution, as evidenced by changes in XPS spectra, elemental composition, and SEM morphology (Figure 5 and Figure 7 and Table 1). The formation of pits and the smoothing of crystal boundaries indicate partial dissolution of the surface crystallites [44]. As we have shown in our previous work [25] this material dissolves in the beginning via a spiral mechanism, where the center of the grain dissolves at a higher rate than the periphery, a behavior typical of crystals with structural defects [44]. As time passes and the concentration of Ca^2+^ ions in the solution increases, the thermodynamic driving force for dissolution decreases, causing the rates to equalize—a process known as self-inhibition. This is driven by calcium-rich Ca_2_(PO_4_)(OH) groups with Ca/P = 2 and adsorbed CO_2_ passing into the solution, alongside the adsorption of water and lactic acid molecules onto the surface [45,46]. This scenario suggests that prolonged contact with the synthetic CHA tablet will lead to thinning rather than significant heterogeneous changes on the surface.

Natural HA, in contrast, exhibits resistance to acidic attack during the early stages of exposure. Detectable structural degradation occurs only after prolonged exposure to the demineralizing solution, and dissolution preferentially occurs along intercrystalline boundaries (Figure 7). This behavior likely reflects the hierarchical microstructure and compositional heterogeneity typical of natural apatites [47]. It seems that natural apatite follows a chemical model of dissolution [44]. In this process, ions are separated in a specific sequence: first hydroxide/fluoride ions, followed by calcium ions, and finally orthophosphate ions [44]. This sequence leads to the formation of virtual intermediate compounds, namely Ca_3_(PO_4_)_2_ and CaHPO_4,_ with a Ca/P ratio between 1.5 and 1, which is consistent with the Ca/P ratio obtained by us (Table 1). Our research shows that, under the experimental conditions, 12 h are sufficient to simulate disruption of the outer CHA layer, analogous to early lesions in tooth enamel, while for natural HA, 6 days of contact are required.

The appearance of Pb in the surface composition of natural HA during demineralization suggests that trace elements become detectable when partial dissolution exposes deeper layers of the structure.

The experiments with the remineralization-active solution reveal surface reconstruction mechanisms for the two materials. XPS spectra (Figure 8) show the formation of new chemical environments associated with Ca–NO_3_/Ca-Cl and Na–O–P bonds, indicating the deposition of newly formed calcium phosphate phases from the solution. The broadening and asymmetry of the peaks, particularly in synthetic CHA, suggest a more heterogeneous surface layer [48] composed of hydrated and carbonate-containing species [Figure 8]. This interpretation is supported by the increase in carbon and oxygen-related surface components observed in the deconvoluted spectra. The elemental analysis (Table 1) indicates that remineralization forms a carbon-rich surface layer on synthetic CHA, originating from the artificial saliva components. The high O/Ca and O/P ratios suggest the presence of hydroxylated and hydrated surface species, typical of early-stage calcium phosphate precipitation. In contrast, natural HA maintains a surface composition closer to the mineral phase, with Ca/P values approaching those of natural enamel.

SEM observations confirm these differences in mineral growth behavior (Figure 9). Synthetic CHA develops a dense, fine-grained layer covering the entire surface, which suggests rapid nucleation and uniform precipitation of small crystallites. In contrast, remineralization of natural HA occurs through the growth of larger crystals preferentially localized in structural defects exposed during the dissolution stage. This localized growth mechanism resembles the natural remineralization processes occurring in enamel, where mineral deposition often initiates at defect sites [49].

The results demonstrate that natural HA exhibits greater chemical stability during demineralization and a more enamel-like response during remineralization, whereas synthetic CHA undergoes more pronounced surface restructuring and forms a highly hydrated, carbonate-rich surface layer.

## 5. Conclusions

The present study provides a comparative investigation of the structural, chemical, and morphological behavior of synthetic and natural non-biogenic (natural mineral) HA under conditions simulating demineralization and remineralization processes in the oral environment.

Both materials exhibit a crystalline apatite structure; however, natural HA contains a small amount of additional fluorapatite component and a broader range of trace elements, reflecting its more complex formation environment. Synthetic CHA is characterized by a greater presence of calcium carbonate or carbonate-substituted species on the surface, while calcium phosphate species dominate the surface of natural HA. These structural and compositional differences strongly influence their surface behavior in acidic and remineralizing conditions.

Exposure to the lactic acid-based demineralizing solution revealed significantly greater resistance of natural HA to chemical attack than that of synthetic CHA. Our research shows that, under the experimental conditions, 12 h are sufficient to simulate disruption of the outer synthetic CHA layer, analogous to early lesions in tooth enamel, while for natural HA, 6 days of contact are required. The two samples also differ in their dissolution mechanisms. Synthetic CHA undergoes faster surface modification, characterized by the formation of pits and changes in surface composition, whereas natural HA shows slower degradation, mainly localized along inter-crystalline boundaries.

The remineralization treatment results in the formation of new surface layers on both materials. XPS analysis indicates the development of new chemical environments associated with Ca–NO_3_/Ca-Cl and Na–O–P bonds and an increased contribution of hydroxyl and organic-related species. Synthetic CHA forms a fine-grained, homogeneous surface layer enriched in carbonate and hydrated components, while natural HA exhibits the growth of larger crystals preferentially within structural defects.

In conclusion, while the newly formed layer does not exhibit an organized crystalline arrangement, its targeted filling of structural defects in natural HA is consistent with the mechanisms of early enamel remineralization.

## Figures and Tables

**Table 1:** Elementary composition on the tablet surfaces from XPS analysis in atomic %.

Sample	Ca	P	O	C	Na	F	N	Pb	Si	Ca/P	O/Ca	O/P
Initial samples												
CHA-synthetic	6.4	2.7	21.5	69.1	0.2	-	-	-	-	2.36	3.3	7.8
HA-natural	7.1	4.7	30.2	53.5	0.5	0.9	2.2	-	0.9	1.51	4.25	6.41
Enamel [36]	2.70	2.05	21.3	68.4	0.30		4.90	-	-	1.31	7.9	10.4
After exposure to a solution with demineralization activity (DE)												
CHA-synthetic 12 h DE	8.6	5.2	33.3	52.3	0.7	-	2.4	-	-	1.65	3.9	6.4
HA-natural 12 DE	8.57	6.29	36.8	39.9	1.80	1.40	2.05	0.2	2.88	1.36	4.29	5.85
HA-natural 6 d DE	5.84	4.22	28.1	55.7	1.81	0.67	2.16	0.26	1.15	1.38	4.81	6.65
After exposure to a solution with remineralization activity (RE)												
CHA-synthetic	3.5	2.1	23.5	70.0	0.9	-	2.3	-	-	1.72	6.7	11.4
HA-natural	7.3	5.8	32.4	49.9	1.43	-	2.36	-	0.85	1.25	5.55	4.46

## Data Availability

The data presented in this study are available in the article.

## Supplementary Materials

The following supporting information can be downloaded at: https://www.mdpi.com/article/10.3390/biomimetics11050338/s1, Figure S1: PXRD powder pattern of the dried at 90 °C precipitate; Figure S2: XPS high-resolution spectra for Ca2p (a,d); O1s (b,e) and C1s (c,f) of the synthetic CHA (a–c) and natural HA (d–f) after 6 h contact with solution containing PCB. P2p spectra are not included as no differences with the initial spectra were observed.
