# Supplementary material for: Comparative Study on the Surface Properties of Synthetic Carbonated Hydroxyapatite and Natural Hydroxyapatite Before and After Contact with Solutions with de- and Remineralization Activity
Source: Biomimetics (Basel). 2026 May 12;11(5):338. doi: 10.3390/biomimetics11050338 (PMC13205011; doi:10.3390/biomimetics11050338)
Supplement: Supplementary file 1 [file biomimetics-11-00338-s001.zip › biomimetics-4258716-supplementary.pdf]

Comparative study on the surface properties of synthetic carbonated hydroxyapatite and natural hydroxyapatite before and after contact with solutions with de- and remineralization activity

Radost Ilieva[1], Ivalina Avramova[1], Ognyan Petrov[2] and Diana Rabadjieva[1*]

[1] Institute of General and Inorganic Chemistry, Bulgarian Academy of Sciences, Acad. G. Bonchev str., bl 11, Sofia 1113, Bulgaria; radipl@mail.bg (RI), iva@svr.igic.bas.bg (IA), didiarab@svr.igic.bas.bg (DR)
[2] Institute of Mineralogy and Crystallography, Bulgarian Academy of Sciences, Acad. G. Bonchev str., bl.107, 1113 Sofia, Bulgaria; opetrov52@gmail.com (OP)
* Correspondence: didiarab@svr.igic.bas.bg (DR)

Supplementary material

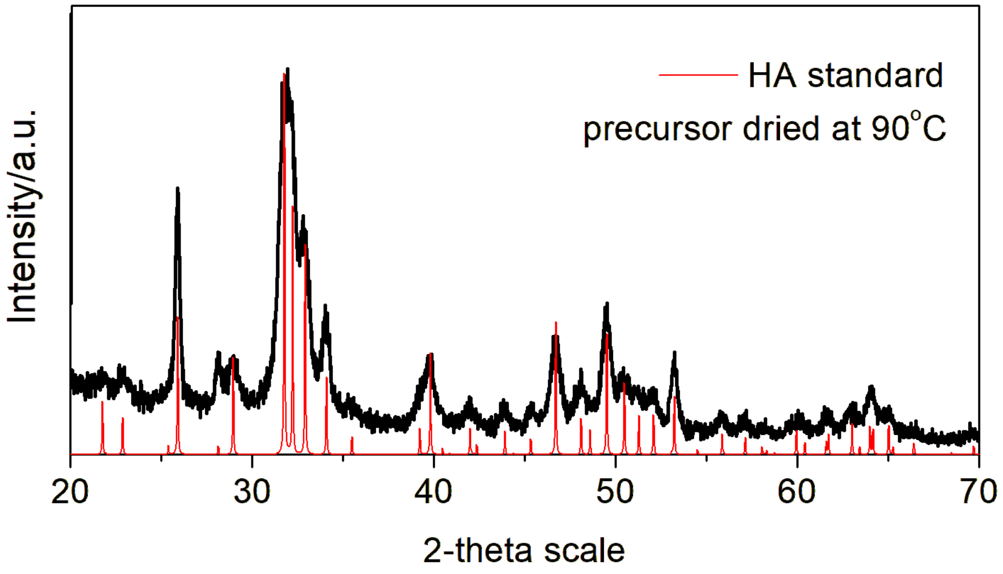

Figure S1 PXRD powder pattern of the dried at 90°C precipitate.

Characteristic reflections of hydroxyapatite are observed; however, they appear as broadened features, particularly in the 31–33° 2θ region. HA standard is ICSD database code 60521

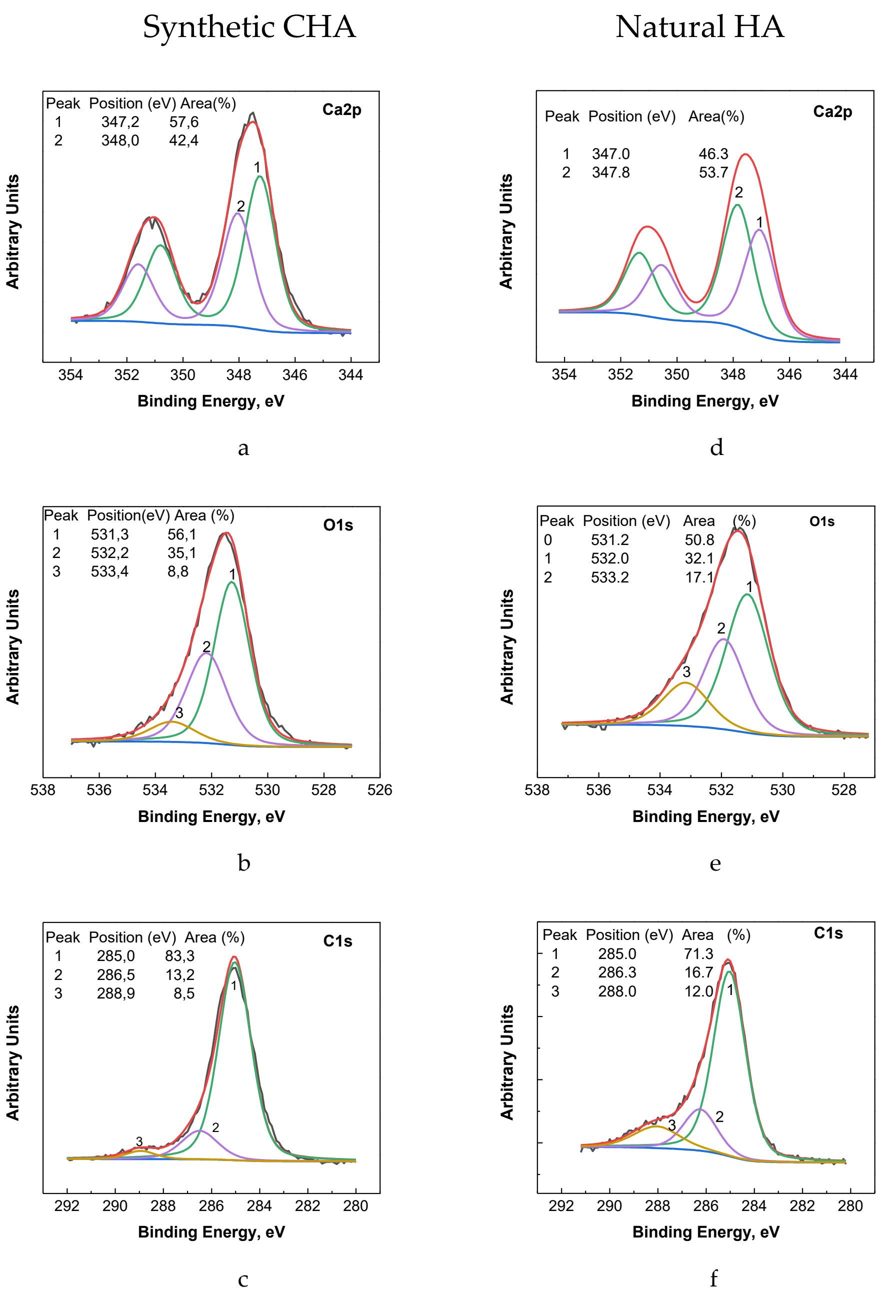

Figure S2. XPS high-resolution spectra for Ca2p (a, d); O1s (b, e) and C1s (c, f) of the synthetic CHA (a, b, c) and natural HA (d, e, f) after 6 h contact with solution containing PCB. P2p spectra are not included as no differences with the initial spectra were observed.

In the Ca2p region the significant difference is observed at Natural HA, where the component associated with carbonate-bound environments increase at the expense of component associated with phosphate environments but the dominance of the latter remains

In both samples, the most significant differences are observed in the increase of C-O species at the expense of absorbed water or CO_3 groups. See peak 2, Figure S2b and S2e compared to peak 2, Figure 5d and 5f for the O1s spectra and peak 2, Figures S2c and S2f compared to peaks 2 and 3, Figure 5g and peak 2 5i.
